# Rapid and Simplified Determination of Amphetamine-Type Stimulants Using One-Pot Synthesized Magnetic Adsorbents with Built-In pH Regulation Coupled with Liquid Chromatography–Tandem Mass Spectrometry

**DOI:** 10.3390/jox15040102

**Published:** 2025-07-02

**Authors:** Yabing Shan, Ying Chen, Jiayi Li, Xianbin Zeng, Rui Jia, Yuwei Liu, Dongmei Li, Di Chen

**Affiliations:** 1 National Narcotics Laboratory Beijing Regional Center, Beijing 100164, China; shanyabing2020@163.com (Y.S.); chenying199810@163.com (Y.C.); jiayilii@163.com (J.L.); zengxianbin133@163.com (X.Z.); jr185190608380@163.com (R.J.); 2 School of Pharmaceutical Sciences, Zhengzhou University, Zhengzhou 450001, China; lyw20250515@163.com

**Keywords:** amphetamine-type stimulants (ATSs), magnetic solid-phase extraction (MSPE), built-in pH regulation, liquid chromatography–tandem mass spectrometry (LC-MS/MS), magnetic adsorbent, one-pot grinding

## Abstract

Background: Amphetamine-type stimulants (ATS) in water pose significant public health and ecological risks, necessitating reliable and efficient detection methods. Current approaches often involve time-consuming pH adjustments and post-processing steps, limiting their practicality for high-throughput analysis. This study aimed to develop a streamlined method integrating pH regulation and adsorption into a single material to simplify sample preparation and enhance analytical efficiency. Methods: A novel Fe_3_O_4_/MWCNTs-OH/CaO composite adsorbent was synthesized via a one-pot grinding method, embedding pH adjustment and adsorption functionalities within a single material. This innovation enabled magnetic solid-phase extraction (MSPE) without pre-adjusting sample pH or post-desorption steps. The method was coupled with liquid chromatography–tandem mass spectrometry (LC-MS/MS) for ATS detection. Optimization included evaluating adsorption/desorption conditions and validating performance in real water matrices. Results: The method demonstrated exceptional linearity (R^2^ > 0.98), low detection limits (0.020–0.060 ng/mL), and high accuracy with relative recoveries of 92.8–104.8%. Precision was robust, with intra-/inter-day relative standard deviations (RSDs) below 11.6%. Single-blind experiments confirmed practical applicability, yielding consistent recoveries (relative errors: 1–8%) for ATS-spiked samples at 0.8 and 8 ng/mL. Compared to existing techniques, the approach reduced processing time to ~5 min by eliminating external pH adjustments and post-concentration steps. Conclusions: This work presents a rapid, reliable, and user-friendly method for ATS detection in complex environmental matrices. The integration of pH regulation and adsorption into a single adsorbent significantly simplifies workflows while maintaining high sensitivity and precision. The technique holds promise for large-scale environmental monitoring and forensic toxicology, offering a practical solution for high-throughput analysis of emerging contaminants.

## 1. Introduction

Amphetamine-type stimulants (ATSs) are a group of psychoactive substances that include amphetamine (A), methamphetamine (MA), and their derivatives [1,2]. These compounds are widely abused due to their stimulant influences on the central nervous system, posing significant health risks and social concerns [3,4]. In addition to their misuse, ATSs are classified as emerging contaminants in environmental matrices, as they can persist in water systems due to improper disposal and human excretion. As a result, it is vital to develop efficient and trustworthy methods for identifying ATSs in both public health and forensic contexts [5,6].

Traditional analytical methods for ATS determination, such as gas chromatography–mass spectrometry (GC-MS) and liquid chromatography–tandem mass spectrometry (LC-MS/MS), are well-regarded for their high sensitivity and specificity. However, due to the low concentrations of target ATS, complex sample matrices, and limited volumes of biological samples, GC-MS and LC-MS/MS alone often struggle to meet the demands of detecting ATSs in rare and challenging samples without proper sample preparation [7,8]. To overcome these obstacles, many techniques for sample preparation have been devised to concentrate the target analytes and purify the samples [9,10]. Various approaches, including liquid–liquid extraction (LLE), liquid-phase microextraction (LPME), dispersive liquid–liquid microextraction (DLLME), solid-phase extraction (SPE), and magnetic solid-phase extraction (MSPE), have been successfully utilized for ATS analysis [11,12].

Among these, MSPE has appeared as a hopeful alternative, offering high efficiency, rapid separation, and operational simplicity, making it particularly well-suited for simplifying the sample preparation process [13,14,15,16]. However, reported MSPE methods for ATS analysis often involve additional pretreatment steps, such as pH adjustments, extensive extraction procedures, and post-desorption processing (e.g., drying and reconstitution) [17,18]. These steps are not only time-consuming and labor-intensive but also prone to variability, which limits their practicality for routine high-throughput applications. Therefore, further advances in MSPE are essential to streamline the workflow and enhance its efficiency.

This research introduced a new magnetic adsorbent with integrated pH control, combined with LC-MS/MS, for the effective measurement of ATSs. The Fe_3_O_4_/MWCNTs-OH/CaO composite adsorbent, synthesized using a one-pot grinding method, integrates magnetic properties, high adsorption efficiency, and inherent pH regulation into a single-step process. This built-in pH regulation feature enables the adsorbent to adjust the sample solution to an optimal alkaline pH during extraction, facilitating ATS adsorption without the need for additional pH adjustment steps. The proposed method was systematically optimized and validated. The method’s robustness, precision, and accuracy were further demonstrated through single-blind experiments with spiked water samples. The method’s benefits were compared with previously reported approaches in terms of processing time, sample preparation complexity, and detection sensitivity to evaluate its potential advantages.

## 2. Materials and Methods

### 2.1. Chemicals and Reagents

**Figure 1:**
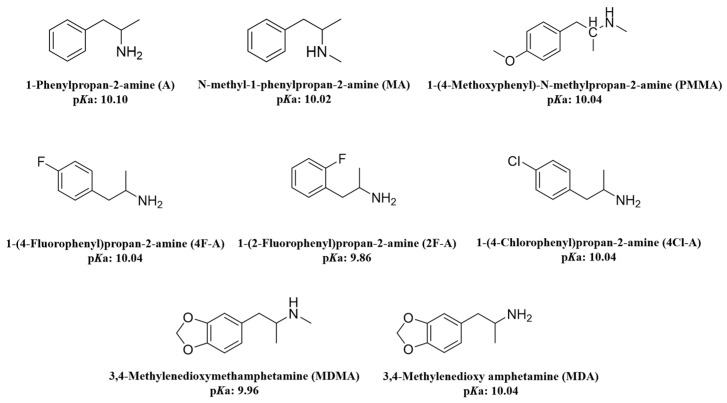
The structures of eight amphetamine-type stimulants (p*K*a values were predicted using ChemDraw 20.0.0.41).

All ATS standards, including 1-phenylpropan-2-amine (A), N-methyl-1-phenylpropan-2-amine (MA), 1-(4-methoxyphenyl)-N-methylpropan-2-amine (PMMA), 3,4-methylenedioxymethamphetamine (MDMA), 1-(2-fluorophenyl)propan-2-amine (2F-A), 1-(4-chlorophenyl)propan-2-amine (4Cl-A), 1-(4-fluorophenyl)propan-2-amine (4F-A) and 3,4-methylenedioxyamphetamine (MDA), were obtained from Shanghai Yuansi Standard Sample Technology Co., Ltd. (Shanghai, China) and were supplied as 1 mg/mL methanol solutions. The structures of these eight ATS standards, along with their p*K*a values (predicted using ChemDraw 20.0.0.41), are shown in Figure 1. The deuterated internal standards (A-d5 and MDMA-d5), intended for use as internal standards, were also sourced from Shanghai Yuansi Standard Sample Technology Co., Ltd. (Shanghai, China) and were provided as 100 μg/mL methanol solutions.

Methanol (MeOH) and acetonitrile (ACN) of LC-MS grade were obtained from Shanghai Anpu Experimental Technology Co., Ltd. (Shanghai, China). Ethanol (EtOH, analytical grade) and formic acid (FA, 98%, MS grade) were sourced from Beijing J&K Scientific Co., Ltd. (Beijing, China). Ultra-pure water (H_2_O) was obtained from Wahaha Group Co., Ltd. (Hangzhou, China). The hydroxyl-functionalized multi-walled carbon nanotubes (MWCNTs-OH, ≥95 wt%) with outer diameters ranging from 30 to 50 nm, inner diameters of 5 to 12 nm, and lengths of 10 to 20 μm were obtained from Aladdin Biochemical Technology Co., Ltd. (Shanghai, China). Fe_3_O_4_ nanoparticles (~50 nm) were sourced from Shanghai MCC New Materials. Aladdin Biochemical Technology Co., Ltd. (Shanghai, China) supplied the calcium oxide (CaO).

### 2.2. Solution Preparation

A mixed standard solution of eight amphetamine-type stimulants (ATSs) was prepared in methanol, with each analyte at a concentration of 0.1 mg/mL. The solution was stored at −20 °C in a light-protected environment and freshly prepared weekly to ensure stability. It was subsequently diluted with water to obtain the desired working concentrations, which were freshly prepared daily to maintain accuracy and reliability.

A working aqueous solution of eight amphetamine-type stimulants was prepared in H_2_O, with each analyte at a concentration of 1 ng/mL. This solution was utilized to assess the efficiency of extraction and optimize the method. The pH adjustments were carefully performed using a Mettler-Toledo SevenDirect SD50 pH meter (Mettler-Toledo, Greifensee, Switzerland) and adjusted through the use of calcium oxide powder.

### 2.3. Sample Collection

The water samples used in this study were collected from the Gulou West Street river section in Beijing (39.94° N, 116.39° E), located at the boundary between urban residential areas and non-motorized traffic lanes. This site is representative of typical surface-water environmental conditions. The basic physicochemical properties of the water samples were as follows: pH 8.0, conductivity 732.8 μS/cm, turbidity 10.9 NTU, dissolved oxygen 7.4 mg/L, and ammonia nitrogen 0.09 mg/L. Samples were collected at a depth of 50 cm below the surface using a polyethylene sampler and transported to the laboratory within 4 h under refrigerated conditions (4 °C). The complete dataset is available on the National Real-time Surface Water Quality Automatic Monitoring and Data Release System of the Ministry of Ecology and Environment of China (https://www.mee.gov.cn/, accessed on 2 March 2025).

### 2.4. Material Preparation

**Figure 2:**
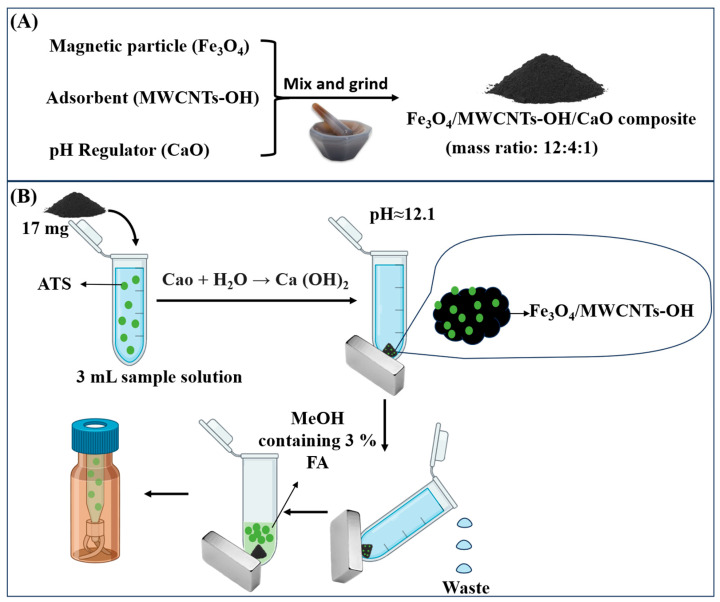
A schematic illustration of (**A**) the preparation of Fe_3_O_4_/MWCNTs-OH/CaO composite and (**B**) the MSPE procedure with integrated pH adjustment for analyte extraction from water samples.

Through a single-step grinding process, a magnetic adsorbent with built-in pH control was developed, following our previously reported method [19,20]. This method combined magnetic nanoparticles made of Fe_3_O_4_ and multi-walled carbon nanotubes with hydroxyl groups (MWCNTs-OH) and calcium oxide (CaO), which served as the pH regulator (Figure 2A). During the preparation process, Fe_3_O_4_, MWCNTs-OH, and CaO were combined in a precise mass ratio of 12 mg:4 mg:1 mg, which represents the optimized conditions established in our previous study [20]. The mixture was then ground thoroughly until a uniform and homogeneous powder was achieved. Through this simple yet effective approach, Fe_3_O_4_, MWCNTs-OH, and CaO spontaneously self-assembled into a stable magnetic material. The final product, named Fe_3_O_4_/MWCNTs-OH/CaO, was specifically designed to perform dual functions: efficient analyte extraction and simultaneous pH regulation. When the material was added to 3 mL of water, the measured pH at room temperature was ≈12.1. The pH regulation mechanism by CaO is as follows: CaO reacts with water to form strongly alkaline Ca(OH)_2_: CaO + H_2_O → Ca(OH)_2_ → Ca^2^⁺ + 2OH^−^.

### 2.5. Extraction Procedure

The extraction operation, as depicted in Figure 2B, utilized the Fe_3_O_4_/MWCNTs-OH/CaO composite as both an adsorbent and a built-in pH regulator. Specifically, 17 mg of the composite was added to 3 mL of sample solution. Upon contact with water, the CaO component in the composite reacted to form Ca(OH)_2_, elevating the solution’s pH to approximately 12.1. The sample was vortex-mixed for 2 min in order to facilitate the adsorption of the analytes onto the composite. The in situ self-assembled magnetic adsorbent (Fe_3_O_4_/MWCNTs-OH), along with the captured analytes, was subsequently isolated by applying an external magnet, allowing the supernatant to be removed and discarded. To eliminate residual impurities, the adsorbent was rinsed with 500 μL of water under vortexing for 30 s. Subsequently, 200 μL of desorption solvent was introduced, and the mixture was vortexed for 2 min to elute the analytes from the adsorbent. After magnetic separation to immobilize the self-assembled Fe_3_O_4_/MWCNTs-OH, the desorbed solution was collected and analyzed using LC-MS/MS.

### 2.6. LC-MS/MS Analysis

The analysis was conducted using a 6495D mass spectrometry system coupled with the 1290 Infinity II UHPLC system (Agilent Technologies, Santa Clara, CA, USA). For the purpose of chromatographic separation, a Waters ACQUITY UPLC CSH C18 column (100 mm × 2.1 mm, 1.7 μm; Waters, Milford, MA, USA) was used. Phase A was water with 0.1% formic acid, while phase B was acetonitrile. The separation was carried out using a gradient elution approach, beginning with 5% B held for 2.0 min, ramping to 30% B over 4.0 min, and further increasing to 95% B at 6.5 min. This condition was maintained at 95% B for 5.5 min, then reduced back to 5% B over 2.0 min, followed by re-equilibration at 5% B for another 2.0 min. The total run time, including column equilibration, was 18.0 min. The flow rate was maintained at 0.3 mL/min. Additionally, the column temperature was set to 30 °C, and the injection volume used was 1 μL.

In positive ion mode, electrospray ionization (ESI) was utilized for ionization. The detection was carried out using multiple reaction monitoring (MRM) mode. The mass spectrometry conditions, including the retention times for each analyte, are detailed in Table 1. The nebulizer gas pressure was 35.0 psi, while the drying and sheath gas flow rates were set to 13.0 L/min and 11.0 L/min, respectively. The drying gas temperature was maintained at 200 °C, while the sheath gas temperature was set to 350 °C. Data acquisition and processing were performed using Agilent MassHunter Workstation software (version 12.2; Agilent Technologies, Santa Clara, CA, USA).

## 3. Results and Discussions

### 3.1. Experimental Design

Previously, our group developed a one-pot synthesis of a magnetic adsorbent with a combined pH control approach for the swift detection of antidepressant compounds in biological fluids [21]. In this approach, Fe_3_O_4_ magnetic particles, hydroxyl-functionalized multi-walled carbon nanotubes (MWCNTs-OH), and calcium oxide (CaO) were combined in a single-step grinding process to create the Fe_3_O_4_/MWCNTs-OH/CaO composite. During extraction, the CaO in the composite reacts with water to form Ca(OH)_2_, directly adjusting the solution’s pH to an alkaline state. Under these conditions, antidepressant molecules predominantly exist in their neutral form, which facilitates their adsorption onto the adsorbent, eliminating the need for external pH adjustments. This simplifies the sample preparation process and enhances the efficiency of antidepressant extraction. This strategy has been proven effective for detecting antidepressants; however, its broader applicability requires further validation.

Amphetamine-type stimulants (ATS) are hydrophobic, basic compounds. ChemDraw (version 20.0.0.41) predictions indicate that their p*K*a values range from 9.86 to 10.10. At pH ≥ 11, all eight ATSs predominantly exist in their neutral molecular forms, making them suitable for adsorption through hydrophobic interactions with the adsorbent. Numerous studies have also reported that adsorbents are most effective at pH 10 or 11 for extracting ATSs [17,22]. Building upon this foundation, the application of Fe_3_O_4_/MWCNTs-OH/CaO composites to the efficient extraction of ATSs from aqueous samples was promising. The built-in pH regulation capability of CaO eliminates the need for external pH adjustment, streamlining the sample preparation process while ensuring optimal extraction conditions for ATSs. Given that ATSs primarily exist in their neutral forms at pH ≥ 11, the in situ formation of Ca(OH)_2_ within the adsorbent provides a favorable environment for their adsorption through hydrophobic and π–π interactions with the MWCNTs-OH component.

### 3.2. Optimization of Extraction Conditions

To achieve optimal extraction conditions, the critical parameters affecting both extraction and desorption performance were thoroughly investigated. These parameters included the amount of Fe_3_O_4_/MWCNTs-OH/CaO composite, the type of desorption solvent, the acid concentration in the desorption solvent, and the desorption solvent volume. A mixed solution containing ATSs at a concentration of 1 ng/mL was utilized for the optimization process. The extraction recovery of the target analytes was used as the key evaluation metric. All experiments were conducted in triplicate (*n* = 3), and the average values were compared to determine the optimal conditions.

#### 3.2.1. Mass of CaO in the Composite

**Figure 3:**
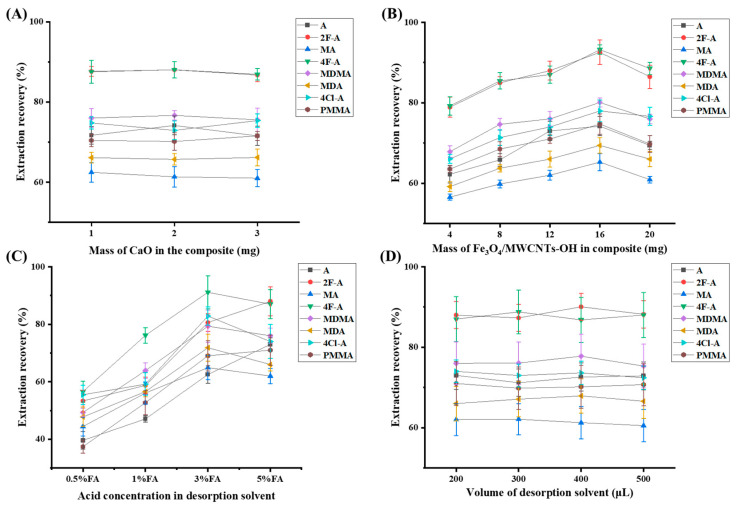
The optimization of the crucial parameters of the proposed method: (**A**) the mass of CaO in the composite; (**B**) the mass of Fe_3_O_4_ and MWCNT-OH in the composite; (**C**) the acid concentration in the desorption solvent; and (**D**) the volume of the desorption solvent.

The amount of CaO in the composite is critically important for the regulation of pH and influences the extraction efficiency of ATSs. To optimize this parameter, different CaO masses (1 mg, 2 mg, and 3 mg) were evaluated, and the corresponding extraction recoveries were compared. As shown in Figure 3A, the extraction recoveries exhibited minimal variation across the tested CaO amounts, indicating that 1 mg of CaO was sufficient to effectively regulate pH. Therefore, to maintain efficiency while minimizing material usage, 1 mg of CaO was chosen as the ideal quantity for follow-up experimental procedures.

#### 3.2.2. Mass of Fe_3_O_4_ and MWCNT-OH in the Composite

Based on our previous experience, a Fe_3_O_4_ to MWCNT-OH mass ratio of 3:1 was found to promote strong magnetic self-assembly and enhance magnetic responsiveness [19,23]. Therefore, this ratio was maintained while optimizing the total mass of Fe_3_O_4_ and MWCNT-OH in the composite. Different total masses (4 mg, 8 mg, 12 mg, 16 mg, and 20 mg) were evaluated, and their extraction recoveries were compared. As shown in Figure 3B, extraction efficiency was insufficient when the total mass was too low, likely due to inadequate adsorption capacity. Conversely, when the mass was too high, desorption became more challenging, reducing overall efficiency. Among the tested conditions, 16 mg (12 mg Fe_3_O_4_ and 4 mg MWCNT-OH) provided the best balance between adsorption and desorption performance. Therefore, 16 mg was selected as the optimal amount for subsequent experiments.

#### 3.2.3. Type of Desorption Solvent

The choice of desorption solvent is a critical factor for effectively desorbing ATSs from the magnetic adsorbent. Based on the interactions between ATSs and the adsorbent, the desorption efficiencies of three commonly used eluents—MeOH, ACN, and EtOH, all containing 5% FA—were systematically evaluated. The addition of 5% FA serves to neutralize any residual undissolved CaO. Additionally, FA converts ATSs into their ionic forms, enhancing desorption efficiency, improving ionization efficiency, and ultimately increasing detection sensitivity. However, when using ACN and EtOH, certain compounds (e.g., PMMA) exhibited peak broadening and even splitting, likely due to solvent effects caused by EtOH’s high elution strength during chromatographic separation [20,24]. In contrast, MeOH with 5% FA provided the best peak shape while maintaining comparable recoveries to the other solvents. Therefore, MeOH containing 5% FA was chosen as the desorption solvent owing to its optimal balance of recovery, peak shape, and minimal solvent effects.

#### 3.2.4. Acid Concentration in Desorption Solvent

After selecting MeOH as the desorption solvent, the effect of different concentrations of FA in the solvent (0.5% FA, 1% FA, 3% FA, and 5% FA) was further optimized. As shown in Figure 3C, the extraction recoveries gradually increased with higher FA concentrations. At 3% FA, the recovery of amphetamine-type stimulants (ATSs) reached its peak. However, increasing the FA concentration beyond 3% did not improve the extraction recovery further. Therefore, a MeOH solution containing 3% FA was selected as the final desorption solvent for its optimal performance.

#### 3.2.5. Volume of Desorption Solvent

The desorption solvent volume was optimized using MeOH containing 3% FA. Volumes ranging from 200 μL to 500 μL were evaluated, with signal intensity (peak area) used as the criterion instead of extraction recovery. As shown in Figure 3D, the results indicated minimal variation in recovery, suggesting that 200 μL is sufficient for effective desorption. This is likely due to the high desorption efficiency of acidic methanol. Given that the desorbed solution was directly injected for analysis in this study, a smaller volume results in a higher analyte concentration, thereby enhancing detection signals and sensitivity. Consequently, 200 μL was selected as the optimal desorption solvent volume.

### 3.3. Performance Evaluation

**Figure 4:**
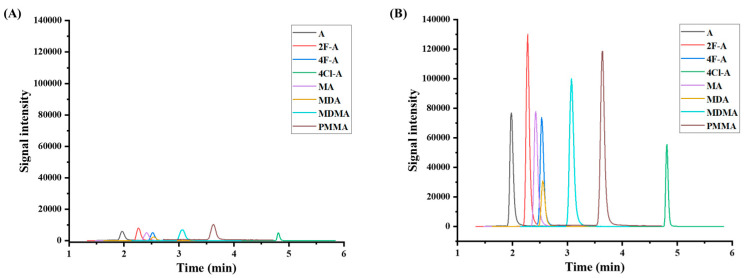
MRM chromatograms of (**A**) ATS standard solution (1 ng/mL) and (**B**) ATS standard solution (1 ng/mL) processed using the proposed sample preparation method.

Under the optimized conditions, the performance of Fe_3_O_4_/MWCNTs-OH/CaO in extracting ATSs was evaluated. The proposed approach was applied to standard aqueous ATS solutions (1 ng/mL), followed by LC-MS/MS analysis. For comparison, a direct LC-MS/MS evaluation was also performed on a 1 ng/mL standard ATS solution. The MRM chromatograms (Figure 4A) display the LC-MS/MS analysis of eight ATSs at 1 ng/mL without pretreatment, while Figure 4B demonstrates the successful identification of the eight ATSs with significantly enhanced signal intensity due to effective enrichment. To further validate the built-in pH regulation strategy, the pH adjustment capability of Fe_3_O_4_/MWCNTs-OH/CaO was assessed. The results indicated that the solution pH reached approximately 12.1, confirming that the built-in pH adjustment functioned as expected. These findings emphasize the possible capabilities of Fe_3_O_4_/MWCNTs-OH/CaO as an effective pH-regulating adsorbent, simplifying the sample preparation process while maintaining high extraction efficiency.

### 3.4. Method Validation

Matrix effects were assessed to evaluate the influence of co-existing substances in water on the ionization efficiency of the target analytes. As shown in Table 2, the absolute matrix effect values ranged from 56.8% to 73.4%, indicating moderate signal suppression for most analytes when using environmental water as the matrix. However, after internal standard (IS) normalization, the matrix effect values were significantly corrected, ranging from 88.5% to 105.2%. Most values were close to 1.00, demonstrating that the use of an appropriate internal standard effectively compensated for matrix-related variability. These findings highlight the necessity of IS correction in quantitative LC-MS analysis, especially when analyzing trace compounds in environmental matrices.

Given the variability in matrix composition across different samples, which may affect extraction efficiency and LC-MS/MS detection, deuterated ATSs were used as internal standards to ensure accurate calibration. These internal standards corrected for variations during solution handling, the extraction process, and LC-MS/MS analysis. The concentration of the internal standards was set at 1 ng/mL. Specifically, A-d5 was used as the internal standard for MA, A, 2F-A, 4F-A, and 4Cl-A, while MDMA-d5 served as the internal standard for MDA, MDMA, and PMMA. Calibration plots were constructed by graphing the ratios of the analytes’ peak areas to their corresponding internal standards versus the concentrations of the analytes. The detection limit (LOD) and quantification limit (LOQ) were established using signal-to-noise ratios of 3:1 and 10:1, respectively.

It was observed that the approach displayed highly linear behavior in the analysis of all ATSs, with calibration curves showing correlation coefficients (R^2^) greater than 0.98 over the concentration range of 0.1–40 ng/mL (Table 3). The limits of detection (LODs) were as low as 0.020–0.060 ng/mL, indicating the high sensitivity of the method. The accuracy of the approach was evaluated by conducting intra- and inter-day studies at three different concentration levels (0.2, 4, and 20 ng/mL) (Table 4). Intra-day relative standard deviations (RSDs) varied between 1.1% and 7.5%, while inter-day RSDs were between 3.5% and 11.6%, indicating excellent repeatability and reproducibility. These results demonstrate the method’s robustness and reliability for routine analysis of ATSs in complex sample matrices. The validation protocol was established according to: ICH Q2(R1) for linearity, precision and accuracy parameters.

### 3.5. Sample Analysis

To address the challenge of obtaining real samples containing all eight substances, a single-blind experimental design was employed to evaluate the performance of the validated method in real-world scenarios [23,25]. In this approach, the analyst conducting the detection was unaware of the spiked concentrations until the analysis was completed. The eight ATSs were spiked into water samples at low and high concentrations of 0.8 ng/mL and 8 ng/mL, respectively. The results showed excellent agreement between the detected and spiked concentrations. Across all spiking levels, the relative error for the eight ATSs ranged from 1% to 8% (Table 5). These findings confirm the method’s robustness and accuracy for analyzing real samples while minimizing bias, demonstrating its practical applicability for detecting these compounds in complex matrices.

### 3.6. Method Comparison

The proposed method demonstrates several advantages when compared with previously reported MSPE methods for ATS analysis (Table 6). One of the key improvements is the significantly reduced processing time, with the total sample preparation requiring only approximately 5 min. This method significantly reduces processing time compared to other methods, which range from 15 to over 12 h due to additional sample pre-treatment and post-processing steps.

Another major advantage of this method is its simplicity. Unlike other approaches, the proposed method does not require pre-adjustment of the sample solution’s pH before extraction. This eliminates the need for additional reagents and steps, streamlining the workflow and reducing potential sources of variability. Furthermore, no post-processing of the desorbed solution, such as nitrogen blow-down concentration or reconstitution, is necessary. This feature further simplifies the preparation process and minimizes the risk of analyte loss during handling.

Additionally, the adsorbent preparation in this study is greatly simplified compared to conventional methods. Many previously reported MSPE and MIP-SPE approaches involve complex pre-synthesis of functionalized magnetic adsorbents, requiring multi-step modifications, high-temperature reactions, or long preparation times. In contrast, the Fe_3_O_4_/MWCNTs-OH/CaO composite in this study is prepared via a straightforward one-pot grinding approach, eliminating the need for complicated synthesis procedures while maintaining high adsorption efficiency and built-in pH regulation.

In terms of sensitivity, the proposed method achieves LODs comparable to or better than most reported methods. Some studies reporting higher sensitivity often achieve this by using larger sample volumes and longer processing times. Similarly, the sensitivity of this method could be further enhanced by increasing the sample volume and thereby improving the enrichment factor. However, in this study, the sensitivity achieved was sufficient to meet the requirements of most analytical applications, so this adjustment was not implemented.

Overall, the method’s combination of reduced processing time, elimination of pre-adjustment and post-processing steps, simplified adsorbent preparation, and competitive sensitivity highlights its practical advantages and its potential for high-throughput analysis.

## 4. Conclusions

This research introduces an innovative and effective approach for detecting ATSs in water samples from the environment through magnetic solid-phase extraction (MSPE) featuring integrated pH control, combined with liquid chromatography–tandem mass spectrometry (LC-MS/MS). The developed Fe_3_O_4_/MWCNTs-OH/CaO composite adsorbent eliminates the need for pre-adjustment of sample pH and post-processing steps, significantly simplifying the sample preparation workflow. The method demonstrated excellent sensitivity, precision, and robustness. The proposed approach also offers substantial advantages in terms of time efficiency, requiring only ~5 min for total sample preparation, which is markedly shorter than most reported methods. The integration of pH regulation into magnetic adsorbent reduces the reliance on external reagents, minimizing variability and improving analytical reliability. These results highlight the practical applicability of this method for the routine high-throughput analysis of ATSs, offering a promising alternative for environmental and forensic toxicology applications. Furthermore, the material’s pH-responsive adsorption mechanism suggests broader applicability for basic and hydrophobic compounds (p*K*a ~8–11), such as cannabinoids and benzodiazepine-type sedatives—highlighting its potential beyond ATSs for addressing other analytically challenging contaminants in water.

## Figures and Tables

**Table 1:** Retention times, parent ions, product ions, and collision energies for the analysis of ATSs and their deuterated analogs (* represents the quantification ion).

Analyte	Retention Time (min)	Parent Ion (m/z)	Product Ion (m/z)	Collision Energy (eV)
A	1.98	136.1	91.1 *	20
			119.1	13
A-*d_5_*	1.96	141.2	124.1 *	14
			93.1	21
2F-A	2.27	154.2	109.1 *	26
			83.1	42
MA	2.42	150.1	91.1 *	30
			119.1	13
4F-A	2.53	154.2	109.1 *	12
			83.0	40
MDA	2.54	180.1	105.1 *	14
			163.0	22
MDMA	3.07	194.2	163.1 *	14
			105.1	25
MDMA-*d_5_*	3.02	199.1	165.1 *	5
			107.1	16
PMMA	3.64	180.1	121.1 *	21
			149.2	13
4Cl-A	4.80	170.1	125.0 *	20
			153.1	11

**Table 2:** Absolute and IS-normalized matrix effects for eight ATSs in spiked environmental water samples.

Matrix Effect	A	2F-A	MA	4F-A	MDA	MDMA	PMMA	4Cl-A
Absolute matrix effect	66.1%	65.3%	56.8%	67.2%	73.4%	73.1%	73.2%	65.6%
IS-normalized matrix effect	104%	103.3%	88.5%	105.2%	104.1%	104.2%	103.4%	102.1%

**Table 3:** Linear range, linear equations, coefficients of determination (R^2^), limits of detection (LOD), and limits of quantification (LOQ) for ATSs.

Analyte	Linear Range (ng/mL)	Linear Equation	R^2^	LOD (ng/mL)	LOQ (ng/mL)
A	0.1–40	Y = 11.978x − 0.879	0.9998	0.020	0.067
2F-A	0.1–40	Y = 23.590x − 1.470	0.9948	0.020	0.067
MA	0.2–20	Y = 18.551x − 1.999	0.9825	0.050	0.167
4F-A	0.1–40	Y = 12.404x − 0.555	0.9932	0.030	0.100
MDA	0.2–20	Y = 0.365x + 0.039	0.9827	0.060	0.200
MDMA	0.1–40	Y = 1.675x − 0.024	0.9998	0.020	0.067
PMMA	0.1–40	Y = 1.901x − 0.044	0.9997	0.020	0.067
4Cl-A	0.2–40	Y = 0.471x + 0.013	0.9963	0.060	0.200

**Table 4:** Intra-day and inter-day recoveries and relative standard deviations (RSDs) for ATSs at three different spiked concentrations.

Analyte	Added (ng/mL)	Intra-Day		Inter-Day (*n* = 3)	
		Recovery (%)	RSDs (%)	Recovery (%)	RSDs (%)
A	0.2	101.7	6.2	96.3	8
	4	100.7	6	98.1	5.5
	20	104.1	7.3	107.9	10
2F-A	0.2	92.8	2.7	99.3	5.7
	4	98	4.1	102.7	4.6
	20	96.4	6.5	102.1	7.7
MA	0.2	99.3	5	100	4.7
	4	100.5	2.4	102.8	3.9
	20	98.5	7.5	105.9	11.5
4F-A	0.2	98.3	3.9	93.4	5.9
	4	102.3	4.6	99.1	6.9
	20	98	6	93	7.5
MDA	0.2	104.8	2.4	102.6	4.6
	4	103.7	3.7	100.8	4
	20	100.8	5	103.4	8.3
MDMA	0.2	97.2	2.2	101	5.6
	4	101.4	1.1	101.4	3.5
	20	95.8	5.4	101.4	7.5
PMMA	0.2	97.5	2.2	102.1	8.8
	4	103.6	3.5	106.2	8.5
	20	99.3	5	104.6	10.8
4Cl-A	0.2	102	3.9	96.6	10.4
	4	104.4	4.8	109	8.3
	20	102.6	4.2	107	11.6

**Table 5:** Detected concentrations (mean ± SD) of ATSs in water samples spiked at 0.8 ng/mL and 8 ng/mL under a single-blind experimental design.

Spiking Level (ng/mL)	Detected Level (ng/mL) (Mean ± SD)							
	A	2F-A	MA	4F-A	MDA	MDMA	PMMA	4Cl-A
0.8	0.78 ± 0.03	0.77 ± 0.04	0.74 ± 0.01	0.85 ± 0.06	0.82 ± 0.03	0.75 ± 0.06	0.74 ± 0.08	0.82 ± 0.03
8	8.13 ± 0.02	8.53 ± 0.07	8.29 ± 0.04	8.44 ± 0.05	7.43 ± 0.07	7.64 ± 0.04	7.64 ± 0.05	7.85 ± 0.02

**Table 6:** A comparison of the proposed method with existing methods for ATS analysis.

Sample	Sample Preparation Method					Detection Technique	LOD (ng/mL)	Ref.
	Method	Magnetic Adsorbent	Pre-Adjustment of Sample pH	Post Processing After Elution	Processing Time			
Wastewater	MSPE	Fe_3_O_4_@nSiO_2_@mSiO_2_@PDA (complex synthesis)	Yes	Yes	>18 min	LC-MS/MS	0.001–0.005	[17]
Urine samples	MSPE	Fe_3_O_4_@nSiO_2_@mSiO_2_@PDA-C18 (complex synthesis)	Yes	Yes	>18 min	LC-MS/MS	0.01–0.1	[15]
Wastewater	MSPE	DES/ZIF-MGO (complex synthesis)	Yes	Yes	>40 min	LC-MS/MS	0.02–1.55	[16]
Wastewater	MSPE	Fe_3_O_4_@PDA (complex synthesis)	Yes	No	>15 min	LC-MS/MS	0.002–0.005	[26]
Urine samples	QuEChERS	/	Yes	Yes	>15 min	GC-MS	360	[27]
Sediments	QuEChERS	/	No	Yes	>12 h	LC-MS/MS	0.0077–0.0299 (ng/g)	[28]
Urine and Wastewater	SPE	MIP	No	No	>40 min	LC-MS/MS	0.05–0.29	[29]
Water	MSPE with built-in pH regulation	Fe_3_O_4_/MWCNTs-OH (simple one-pot grinding)	No	No	~5 min	LC-MS/MS	0.02–0.06	Present work

DES/ZIF-MGO: Deep eutectic solvent-functionalized magnetic graphene oxide/metal–organic framework nanoparticles; Fe_3_O_4_@PDA: Polydopamine-coated magnetic nanoparticles; Fe_3_O_4_@nSiO_2_@mSiO_2_@PDA-C18: Polydopamine and C18 dual-functionalized magnetic core–shell mesoporous silica; Fe_3_O_4_@PDA: Polydopamine-coated magnetic nanoparticles; MIP: molecularly imprinted polymers.

## Data Availability

The original contributions presented in this study are included in the article. Further inquiries can be directed to the corresponding authors.

## Abbreviations

The following abbreviations are used in this manuscript:

ATS	Amphetamine-type stimulants
A	1-phenylpropan-2-amine
MA	N-methyl-1-phenylpropan-2-amine
GC-MS	Gas chromatography-mass spectrometry
LC-MS/MS	Liquid chromatography–tandem mass spectrometry
LLE	Liquid–liquid extraction
LPME	Liquid-phase microextraction
DLLME	Dispersive liquid–liquid microextraction
MSPE	Magnetic solid-phase extraction
PMMA	1-(4-methoxyphenyl)-N-methylpropan-2-amine
MDMA	3,4-methylenedioxymethamphetamine
2F-A	1-(2-fluorophenyl)propan-2-amine
4Cl-A	1-(4-chlorophenyl)propan-2-amine
4F-A	1-(4-fluorophenyl)propan-2-amine
MDA	3,4-methylenedioxyamphetamine
FA	Formic acid
MeOH	Methanol
ACN	Acetonitrile
EtOH	Ethanol
ESI	Electrospray ionization
MRM	Multiple reaction monitoring
